# Online Sensor Drift Compensation for E-Nose Systems Using Domain Adaptation and Extreme Learning Machine

**DOI:** 10.3390/s18030742

**Published:** 2018-03-01

**Authors:** Zhiyuan Ma, Guangchun Luo, Ke Qin, Nan Wang, Weina Niu

**Affiliations:** 1 School of Computer Science and Engineering, University of Electronics and Technology of China, Chengdu 611731, China; yuliar3514@gmail.com (Z.M.); qinke@uestc.edu.cn (K.Q.); niuweina1@126.com (W.N.); 2 School of Information Science and Engineering, East China University of Science and Technology, Shanghai 200237, China; wangnan@ecust.edu.cn; 3 School of Cybersecurity, Chengdu University of Information Technology, Chengdu 610225, China

**Keywords:** gas sensor, drift compensation, domain adaptation, online learning, extreme learning machine

## Abstract

Sensor drift is a common issue in E-Nose systems and various drift compensation methods have received fruitful results in recent years. Although the accuracy for recognizing diverse gases under drift conditions has been largely enhanced, few of these methods considered online processing scenarios. In this paper, we focus on building online drift compensation model by transforming two domain adaptation based methods into their online learning versions, which allow the recognition models to adapt to the changes of sensor responses in a time-efficient manner without losing the high accuracy. Experimental results using three different settings confirm that the proposed methods save large processing time when compared with their offline versions, and outperform other drift compensation methods in recognition accuracy.

## 1. Introduction

Sensor technologies aim at providing a convenient and intelligent life for human beings and have been largely enhanced in recent years. For example, wearable sensors make health monitoring and early disease classification with minimum discomfort possible [1,2], optical sensors used in clinical diagnosis have made the detection of specific compound such as calcium more easy-to-use [3], and motion sensors have made smart phones not only tools for communications, but also means to provide personalized services [4].

By using specific sensors, identification of chemical gases becomes possible. Electronic-Nose (E-Nose) systems, also known as machine olfaction, is one of such units. Combining with pattern recognition techniques, E-nose systems can classify multiple gases mixed together in different concentrations [5], which leads to a wide application in airport and train station checkpoints [6], food security [7], environmental monitoring [8], clinical diagnosis [9] and so on. Despite the fascinating applications that make our daily lives more intelligent, there has been a major problem in E-Nose systems that makes the recognition capability of sensors degrade after some time. In E-Nose systems, the readings *X* rely on the chemical reactions between gas compounds and the sensor materials, together with some recognition mechanism such as machine learning to create connections between diverse gas types and their corresponding readings. Mathematically, we can use a function y=f(X) to denote such connections. Given a proper trained f(X), the outputs should match all designated gas compounds. However, in practice, when a f(X) is trained on the collected data perfectly, the outputs gradually fail to match the right gases, and the phenomenon is called sensor drift.

Currently, it is commonly accepted that the drift problem in sensors is due to two causes. One is the chemical process that happens between sensor materials and the environment, also called the first-order drift, and the other is the system noise, namely the second-order drift [10,11,12]. Researchers have been trying to solve the problem in material science, sensor selection strategies and post processing mechanisms. In material science, durable materials were invented to prolong the life of sensors [13,14,15,16]. Meanwhile, proper selection of more resilient sensors to drift is another way to achieve the goal [17,18]. In the perspective of post-processing, the drift problem can be taken as the changes of distributions of gas labels over time. To maintain the stable recognition capability of the sensors, classifier ensemble techniques have relived the problem to some extent [19,20,21]. However, the learning process of the methods is supervised and requires human effort to label the training set beforehand. Furthermore, the methods assume that the data distributions remain the same for different gases, which is not always true. Component Correction (CC)-based methods [22,23,24] and Sequential Minimal Optimization (SMO)-based [25,26] are the most effective supervised ways to adjust the model to the drift. Nevertheless, CC-based methods assume the drift acts in the same way for diverse gases, which is sometimes not the case, and SMO-based methods sometimes update the model by following the wrong reference label. Another effective and promising approach is to use the transfer learning technique, namely domain adaptation. Zhang et al. [27] achieved one of the highest accuracies using semi-supervised methods, but the learning mechanism follows an offline training scheme, which makes the data generated in real time hard to be processed in time. Together with the fast development in big data, an enormous amount of sensor data are generated per second, which has made timely processing a great challenge.

To effectively discover information collected from the data, an online learning mechanism that can train and update the model in a time-efficient manner without losing the classification accuracy is required. An online learning method in machine learning uses the current model and newly received data to update the analytical model. In this way, the updates of the model do not require training from scratch and the capability of drift compensation can keep up with the data generated in massive amounts. In this paper, we combine the theory of online sequential extreme learning machine and Domain Adapation Extreme Learning Machine (DAELM) [27] for it has appealing performance and has achieved almost 100% for specific datasets. We wish to transform the offline learning version in the work into an online learning version without losing the performance for drift compensation. The contributions are three-fold:

- The selection of representative samples plays an essential part in semi-supervised methods such as DAELM-S and DAELM-T in [27]. Therefore, we analyzed the characteristics of sample selection and provided two online sampling strategies regarding whether testing error can be used as feedback.
- To preserve the high accuracies of domain adaptation-based methods and save time for updates, we combined the theory of online sequential extreme learning machine to propose Online Source Domain Adaptation Extreme Learning Machine (ODAELM-S). In ODAELM-S, only the source domain and few labeled samples contribute to the model. When new labeled data are identified, ODAELM-S can update the model in a time efficient manner.
- To leverage between the effects of labeled and unlabeled samples, we transformed DAELM-T into its online learning version and proposed Online Target Domain Adaptation Extreme Learning Machine (ODAELM-T). Unlike ODAELM-S, which only relies on the source domain and the labeled set, ODAELM-T leverages the effects of labeled and unlabeled set to the model. Based on the changes of the two sets, the update phase is divided into three learning process, namely unlabeled incremental learning, unlabeled decremental learning and labeled incremental learning.

The remainder of the paper is organized as follows. Section 2 introduces some preliminaries on online processing in sensors, domain adaptation and extreme learning machine to help understand the methods in the paper. Methodologies of ODAELM-S and ODAELM-T are detailed in Section 3. Section 4 describes the dataset used in the experiments and the experimental set-up, followed by a detailed analysis on the results. Conclusions are drawn in Section 5. For illustration purposes, abbreviations of the frequently used terms are listed after Section 5.

## 2. Preliminaries

### 2.1. Online Processing in Sensors

In 2013, Paniagua et al. focused on delivering time efficient drift countermeasures using pattern recognition methods, which is one of the earliest research articles in online drift compensation [28]. However, the methods used in the paper were limited and not much work was done to change the algorithms into online learning versions. Ghafarinia et al. combined pattern recognition with the transient features of Capillary-attached Gas Sensor and proposed an online gas diagnosis algorithm that could determine unknown gases and their concentrations [29]. Nevertheless, the neural network model used in their paper was simple, which consisted of less than 10 nodes, and the features are no more than 40. Moreover, the method was designed for gas diagnosis, instead of drift compensation. With the developments in machine learning, new models and theories have been used for drift problem. However, most of the methods focus on an offline learning manner which leaves online detection and recognition a void. Due to the learning mechanism, some of the methods cannot be simply applied to the scenario. Meanwhile, the time cost for training the model is not trivial due to the growing number of data.

As early as in 2004, Ma et al. introduced the challenges and problems in building online recognition model, although the discussion focused more on the nature of the data rather than the learning algorithms and specific problem domain as sensor drift [30]. In recent years, online processing have been a common issue in sensor related domains. In 2012, Munir et al. proposed an online optimization method to help sensor parameter adjust to the environment changes [31]. In 2013, Zhang et al. proposed online detection for outliers in wireless sensor network to ensure the high quality of the data [32]. Between 2015 to 2017, researches related to online processing in sensors has expanded from decentralized model for resource limited environment [33] and time-efficient monitoring and detection [34,35] to more complicated tasks such as gesture recognition [36], source location [37] and fault diagnosis [38] in specific applications. Therefore, it is also imperative to combine advanced algorithms with online processing for gas sensor drift compensation.

### 2.2. Domain Adaptation

In domain adaptation, the distributions of samples in the data are referred to as domains [39]. In practice, a pre-trained model on a given training set (source domain) usually fails to perform well in testing sets (target domains), due to the distribution differences between training and testing sets. The phenomenon is called domain shift. To deal with the problem, some related approaches include transfer learning, semi-supervised learning, self-taught learning and multiview analysis. In the applications such as computer vision, sentimental analysis, natural language processing, video concept and wifi location detections, domain adaptation techniques have received fruitful results [40,41,42].

In the perspective of transfer learning, domain adaptation is also taken as a special case, in which labeled samples are only available in source domain and both target and source domains share the same single task [43]. Currently, there are four types of methods in domain adaptation, namely instance weighting, self labeling, feature representation and latent feature learning. Due to the expensive efforts for manually labeling the samples in the target domain, feasible and common approach is to perform semi-supervised learning which leverages the effects of limited labeled and unlabeled samples [39,43].

### 2.3. Extreme Learning Machine

As a special type of random neural network, Extreme Learning Machine (ELM) can be represented by a three layer feed forward neural network whose parameters between input and hidden layers are randomized [44]:

(1) $$f_{L}\left(X\right)=\sum_{i=1}^{L}\beta_{i}g_{i}\left(X\right)=\sum_{i=1}^{L}\beta_{i}g\left(a_{i},b_{i},X\right).$$

Specifically, ELM can be written as Equation (1), where $\beta_{1},\beta_{2},\ldots ,\beta_{L}$ are the output weights between the hidden layer with *L* neurons and the output layer, and $g_{i}$ is a nonlinear piecewise continuous function, namely activation function [45]. The output weights of ELM can be acquired by minimizing the approximation error, i.e., Equation (2), where *H*, denoted by Equation (3), is the output of hidden layer with *L* neurons, *n* is the number of neurons in the output layer, and *T* is the target vector of training data:

(2) $$\min_{\beta \in R^{L\times n}}{∥H\beta -T∥}^{2}.$$

Unlike other neural network approaches, ELM directly calculates the output weight matrix β using Equation (4), where $H^{†}$ is the Moore–Penrose pseudoinverse of *H*:

(3) $$H=\left[\begin{array}{ccc} g(a_{1},b_{1},x_{1}) & \cdots & g(a_{l},b_{L},x_{1})\\ g(a_{1},b_{1},x_{2}) & \cdots & g(a_{l},b_{L},x_{2})\\ \vdots & \vdots & \vdots \\ g(a_{1},b_{1},x_{n}) & \cdots & g(a_{l},b_{L},x_{n}) \end{array}\right],$$

(4) $$\beta =H^{†}T.$$

The learning mechanism of classic ELM is tagged as batch learning, which requires a full set of training data. In contrast to batch training, Online Sequential Extreme Learning Machine (OSELM) was proposed to learn from data in a one-by-one or block-by-block way [46]. Instead of using all the samples in the training stage, which is also known as offline learning, OSELM sequentially learns from the data to capture the varying patterns hidden beneath them. In OSELM, the calculation of output weight matrix, denoted by $\beta_{k+1}$, relies only on the new sample $x_{k+1}$ and the previous output weight matrix $\beta_{k}$:

(5) $$\beta_{k+1}=\beta_{k}+P_{k+1}H_{k+1}^{T}\left(T_{k+1}-H_{k+1}\beta_{k}\right).$$

The training process can be formulated as Equation (5), where $P_{k+1}=P_{k}-P_{k}H_{k+1}^{T}{\left(I+H_{k+1}P_{k}H_{k+1}^{T}\right)}^{-1}H_{k+1}P_{k}$, $H_{k+1}$ is the hidden layer output corresponded to the new sample $x_{k+1}$, and $T_{k+1}$ is the corresponding target vector.

## 3. Online Domain Adaptation Extreme Learning Machines

To achieve timely processing without losing the recognition accuracies for sensor drift, we intend to transform current state-of-art batch learning methods into their corresponding online versions. Domain adaptation-based drift compensation has been proved to possess high accuracies in [27]. The two algorithms, namely DAELM-S and DAELM-T, are based on batch learning and require selecting a group of representative samples beforehand. The selection algorithm is based on the distribution of the entire dataset. However, in an online processing scenario, samples in the target domains arrive in sequence as a data flow. Therefore, the original sample selection method is not applicable. Supposing we can determine when to select the representative samples, there is another problem of how much time and human effort it costs. Additionally, the batch learning mechanisms of DAELM-S and DAELM-T require calculating the classifier based on the full dataset. Since the data arrives in sequence, there is no doubt that the data used in the next update will overlap with the ones in previous updates. In this case, there will be repeated calculation of the same data over time, which costs more time and resources as the size of target domain increases.

**Figure 1:**
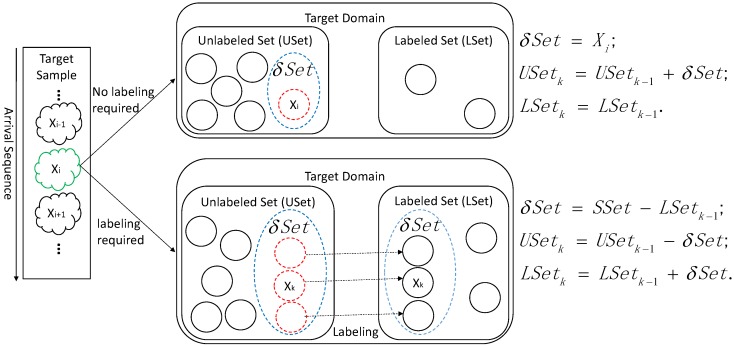
Demonstration of sample changes in the target domain during online processing. SSet is the selected sample in the target domain received to date based on some sample selection algorithm, and δSet is the incremental (decremental) set in labeled (unlabeled) set. For arrival sample $X_{i}$, two cases regarding whether to perform selection and labeling is given in the figure.

In an online learning model using either DAELM-S and DAELM-T, we wish to maintain the performances of the two methods while solving the obstacles described in the previous paragraph. Figure 1 shows the sample changes in a target domain when a new sample $X_{i}$ arrives. The labeled set refers to the manually labeled samples and the unlabeled set is the rest of the samples in a target domain. When $X_{i}$ arrives in the target domain, it triggers two possible cases regarding whether to select and label a group of samples in the unlabeled set. When no selection and labeling happens, $X_{i}$ is added to the unlabeled set and the labeled set remains unchanged. In this case, the changes of samples, denoted by δSet, has only one sample, namely $X_{i}$. When the selection and labeling happens, the situation is more complicated. Given some sample selection algorithm, a group of representative samples, denoted by SSet, is selected from the target samples received to date. Note that, in this case, the unlabeled set includes the new sample $X_{i}$. Considering the fact that the selected samples may overlap with current labeled ones, δSet is the difference set between SSet and $LSet_{k-1},$ where $LSet_{k-1}$ is the latest selected sample set. The blue dashed circle in the figure represents the δSet. When labeling happens, δSet is removed from USet and added into LSet. The $X_{k}$ in δSet refers to any possible samples selected, including the new sample $X_{i}$.

In classic ELM, a variant called OSELM can be applied to reduce the calculation when new sample arrives in sequence. The similar process can be applied to both DAELM-S and DAELM-T by using incremental learning. The idea is to transform original batch learning process into a recursive process. Taking the arrival sequence in Figure 1, for example, when $X_{i-1}$ arrives, the classifier trained on current received data is written as $f(\beta_{T}^{k-1}),$ where $\beta_{T}^{k-1}$ is the parameter or parameter vector that requires training. The output of *f* can be the gas labels or the probabilities of the sample belonging to certain gases. In incremental learning, we wish to derive a recursive form of the training process where $\beta_{T}^{k}$ can be written as a new formula denoted as $g(\beta_{T}^{k-1},K),$ where *K* is some intermediate results. In this way, instead of training the new $\beta_{T}$ with all the data received to date, we can use only the previous result and the current increment for updates. By doing so, we can save the repeated calculation and achieve a time-efficient algorithm for generating new classifiers. In OSELM, the parameter $P_{k+1}$ in Equation (5) is the intermediate result. In DAELM-S and DAELM-T, we can find similar terms to achieve the goal. Once the update algorithm is produced, the only challenge becomes selecting the representative samples in an online manner.

In the following part of the section, we first analyze the sampling strategies regarding online learning. Then, corresponding online learning versions of DAELM-S and DAELM-T are described in detail.

### 3.1. Online Sampling Strategies

Assuming we can determine the time for updates, the only problem is to determine δSet. We view the classification model as an intelligent agent and the arrival sequence of the target samples like perceptions of the environment. Similar to the description on intelligent agents by Stuart and Peter in [47], the actions of the model, including whether to select and label samples, depend on the entire sequence of samples received to date, not on anything that has not yet appeared. Therefore, we can apply Kennard–Stone (KS) algorithm [48,49] on the current received samples to determine δSet. Ideally, if we can perform KS whenever the distribution changes, the problem is solved. However, the changes in distribution may be a slow process, which brings another problem of defining the changes of distribution. Even though a periodical collection of the samples may be a trade-off plan, the circle for collection may vary, which brings up another problem of how to decide the circle beforehand.

Normally, we wish to label enough samples so that the model can be more precise. However, manually labeling in semi-supervised methods is time-consuming and labeling more samples contradicts with our goal of saving time. Eventually, the problem becomes a trade-off between high classification accuracy and low human efforts.

Selecting and labeling samples in a predefined circle is the easiest to apply, although not very applicable since proper circles for different datasets may vary. The reason for labeling more samples is to provide more information to track the changes of data. In this sense, the labeling should be more likely to happen when the performance degrades and vice versa. In this case, we consider the labeling process as a probability event, which possesses the following characteristics:

- The chance of labeling is inversely proportional to number of labeled samples;
- The chance of labeling is proportional to number of samples in total;
- The chance of labeling is proportional to the classification error.

The performance of the model is based on the classification accuracy of current model. In this case, the system would require receiving the accuracy. When the performance of the classification model is assumed to degrade, the labeling should be more frequent, and vice versa. Meanwhile, when the number of labeled samples is small, the labeling should happen more frequently, and vice versa. However, when the accuracy is not accessible, the system would have no idea whether it performs well or not. In this case, the system can only decide for selection and labeling based on the number of samples received so far.

Ideally, if we can determine the representative samples for each target domain as [27] does, we can achieve equivalent high classification accuracies in an online manner. However, the method used to determine the representative samples in [27] is based on the distribution of the entire target domain, which is unable to be acquired in an online scenario. A more feasible way is to label more samples instead of labeling specific ones. Therefore, we use Equation (6) to depict the probability of labeling where ϵ is current classification error, and *y* and *x* are the numbers of labeled samples and entire samples, respectively. In the experiments, the equation managed to possess the aforementioned features while maintaining the number of the labeled samples in a relatively small value. Note that Equation (6) is an attempt to depict the probabilities instead of ideal calculation of the probability. Therefore, more sophisticated methods can be used to replace it:

(6) $$P=\left\{\begin{array}{cc} {\left(1-\frac{y}{x}\right)}^{y}\epsilon , & \epsilon \;is\;accessible,\\ {\left(1-\frac{y}{x}\right)}^{y}, & \epsilon \;is\;not\;accessible. \end{array}\right.$$

In the experiments, the equation is not good enough. Another possible issue may be that the labeled samples keep growing even when the accuracy is high, say over 90%. In an online learning scenario, if the manually labeling process labels too many samples, the method is not applicable since too much resource time would be spent in the process. Therefore, we set another criteria for the process, namely the minimum accepted accuracy. In practice, when the accuracy is stable and very high, it does not require extra labeling. In this paper, the maximum number of KS is set to 50 so as to limit the growth of labeled samples. The minimum accepted accuracy is set to 90%. When the residual error is larger than 10%, the labeled process happens.

### 3.2. Online Source Domain Adaptation Extreme Learning Machine

Similar to OSELM, which uses incoming data to update the model in an online manner, we transformed the training of DAELM-S into an incremental learning procedure and proposed Online Source Domain Adaptive Extreme Learning Machine (ODAELM-S).

**Figure 2:**
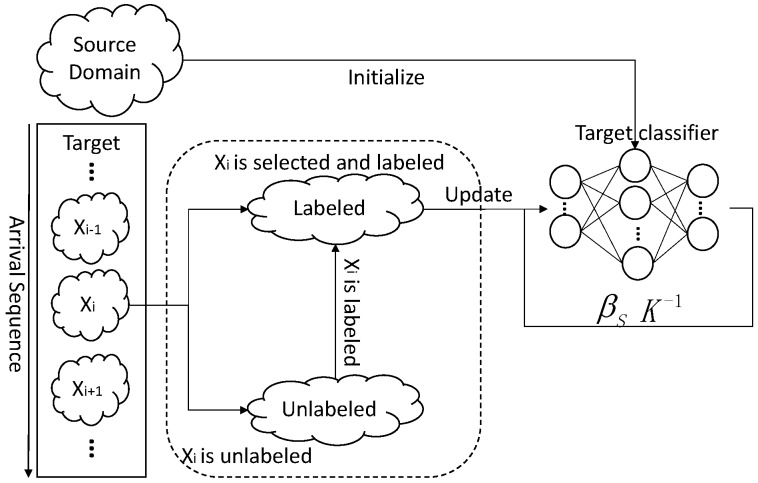
Online updating framework of ODAELM-S.

Figure 2 shows the framework of ODAELM-S. In DAELM-S, the learning only involves the source domain and the labeled samples from target domain. Since the source domain remains unchanged during the learning phase, the update of the model only happens when new samples are labeled. Initially, the target classifier is the source classifier for there is no labeled sample in the target domain. After the classifier is initialized using source domain samples, it can receive and learn the patterns from target domain in an online manner. The left rectangle in the figure represents the arrival sequence of the samples in the target domain. Let $X_{i}$ be the current arrival sample that belongs to either unlabeled or labeled samples. If no selection happens, $X_{i}$ is added into the set of unlabeled samples and there is no update in the classifier. When the online sample selection happens, the sample(s) for labeling can only be chosen from the unlabeled samples and $X_{i}$. Note that $X_{i}$ may not be chosen when the selection happens at the arrival of the sample, but it may be selected later by another selection process. Whenever labeled samples are selected, the target classifier updates itself based on an online learning mechanism described in the following paragraphs.

In DAELM-S, the model is taken as an extension of the classifier trained on the source domain. The objective function is written as Equation (7) where $\epsilon_{S}=H_{S}\beta_{S}-T_{S}$, $\epsilon_{T}=H_{T}\beta_{S}-T_{T}$. $H_{S}$ and $H_{T}$ are the corresponding hidden layer outputs of source domain and labeled samples from target domain, respectively. To obtain an proper value of $\beta_{S}$, it requires minimizing the objective function:

(7) $$L=\;\frac{1}{2}∥\beta_{S}{∥}^{2}+\frac{C_{S}}{2}∥\epsilon_{S}{∥}^{2}+\frac{C_{T}}{2}{∥\epsilon_{T}∥}^{2}.$$

By calculating the gradient of *L* with respect to $\beta_{S}$ as Equation (8), we can calculate the optimal value of $\beta_{S}$ by setting the gradient to 0:

(8) $$\frac{\partial L}{\partial \beta_{S}}=\left(I+C_{S}H_{S}^{T}H_{S}+C_{T}H_{T}^{T}H_{T}\right)\beta_{S}-\left(C_{S}H_{S}^{T}T_{S}+C_{T}H_{T}^{T}T_{T}\right).$$

Note that, regarding whether there are more rows or columns in $H_{S}$, solving $\beta_{S}$ can be either an overdetermined or under-determined problem. When it is an overdetermined problem, we assume $\beta_{S}$ is a linear combination of the columns of $H_{S}$, i.e., $\beta_{S}=H_{S}^{T}\alpha$. Therefore, by setting Equation (8) to 0, the hidden layer output $\beta_{S}$ can be formulated as Equation (9), where $P=H_{S}H_{S}^{T}$ and $Q=H_{S}H_{T}^{T}$:

(9) $$\begin{array}{cc} \beta_{S}= & \left\{\begin{array}{c} {\left(I+C_{S}H_{S}^{T}H_{S}+C_{T}H_{T}^{T}H_{T}\right)}^{-1}\left(C_{S}H_{S}^{T}T_{S}+C_{T}H_{T}^{T}T_{T}\right),\;H_{S}\;has\;more\;rows,\\ H_{S}^{T}{\left(I+C_{S}P+C_{T}P^{-1}QQ^{T}\right)}^{-1}\left(C_{S}T_{S}+C_{T}P^{-1}QT_{T}\right)\;H_{S}\;has\;more\;columns. \end{array}\right. \end{array}$$

Let the case where $H_{S}$ has more rows be case 1 and the other be case 2. When new samples are labeled, the hidden layer output becomes $H_{T}^{k+1}=\left[\begin{array}{c} H_{T}^{k}\\ \delta h \end{array}\right]$ where δh is the corresponding hidden layer output of the newly labeled samples. In order to save the calculation, ODAELM-S uses some intermediate result as $K^{-1}$ where *K* is defined as $I+C_{S}H_{S}^{T}H_{S}+C_{T}H_{T}^{T}H_{T}$ for case 1 and $I+C_{S}P+C_{T}P^{-1}QQ^{T}$ for case 2.

For case 1, $K_{k+1}$ can be defined as Equation (10):

(10) $$K_{k+1}=K_{k}+C_{T}\delta h^{T}\delta h.$$

Hence, $K_{k+1}^{-1}$ can be updated using Equation (11):

(11) $$K_{k+1}^{-1}=K_{k}^{-1}-C_{T}K_{k}^{-1}\delta h^{T}{\left(I+C_{T}\delta hK_{k}^{-1}\delta h\right)}^{-1}\delta hK_{k}^{-1}.$$

Subsequently, $\beta_{S}$ can be updated using Equation (12):

(12) $$\beta_{S}^{k+1}=K_{k+1}^{-1}\left(Right_{k}+C_{T}\delta h^{T}\delta t\right)=\beta_{S}^{k}-C_{T}K_{k+1}^{-1}\delta h^{T}\left(\delta h\beta_{S}^{k}-\delta t\right).$$

For case 2, let $K_{k+1}$ be defined as Equation (13):

(13) $$K_{k+1}=K_{k}+C_{T}P^{-1}H_{S}\delta h^{T}\delta hH_{S}^{T}.$$

Similarly, the update of the intermediate result and output weight can be written as Equations (14) and (15), respectively, where $\delta k=\delta hH_{S}^{T}$:

(14) $$K_{k+1}^{-1}=K_{k}^{-1}-K_{k}^{-1}C_{T}P^{-1}{\left(I+\delta k^{T}\delta kK_{k}^{-1}C_{T}P^{-1}\right)}^{-1}\delta k^{T}\delta kK_{k}^{-1},$$

(15) $$\beta_{S}^{k+1}=H_{S}^{T}K_{k+1}^{-1}Right_{k+1}=\beta_{S}^{k}-C_{T}H_{S}K_{k+1}^{-1}P^{-1}H_{S}\delta h^{T}\left(\delta h\beta_{S}^{k}-\delta t\right).$$

The pseudo code for ODAELM-S is shown in Algorithm 1. Before the updates begin, ODAELM-S first initializes a base classifier using source domain data (lines 1–3). When a base classifier has been created, the classification of gases can be available. When a new sample in the target domain arrives, ODAELM-S calculates the possibility for samples in the target domain to be selected and labeled (lines 5–6). When the process is determined (line 7), a group of unlabeled sample will be selected as δSet (lines 8–9). Based on whether $H_{S}$ has more rows or columns, ODAELM-S updates the corresponding hidden layer output and some intermediate result (lines 10–16). The process continues when no more samples arrive.

**Algorithm 1** Pseudo Code for ODAELM-S.**Input:**  L:= the number of hidden layer neurons;  Act:= the activation function type;  SD:= the source domain data; 1: Initialize two empty sets, i.e., LSet and USet, as labeled and unlabeled sets, respectively; 2: Set activation function as Act and initialize an ELM with *L* hidden nodes with SD; 3: Let Hs be defined as in Equation (9); 4: **while** new sample *x* in the target domain arrives **do** 5: Calculate the probability *P* for labeling; 6: Generate random value between 0 and 1 as *p*; 7: **if**
p<P
**then** 8:  Add *x* to USet; 9:  Select a group of samples from USet as δSet for labeling;10:  **if**
$H_{S}$ has more rows **then**11:   Update the classifier using Equations (11) and (12);12:  **else**13:   Update the classifier using Equation (14) and (15);14:  **end if**15:  Set LSet=LSet−δSet and USet=USet−δSet;16: **else**17:  Add *x* to USet;18: **end if**19: **end while**

### 3.3. Online Target Domain Adpatation Extreme Learning Machine

To transform DAELM-T into its online learning version, we proposed Online Target Domain Adaptation Extreme Learning Machine (ODAELM-T). Different from ODAELM-S, ODAELM-T leverages both labeled and unlabeled samples in the target domain by using Equation (16), in which $\beta_{T}$ is the output weight matrix, $C_{T},H_{T},t_{T}$ are the same as in DAELM-S, and $C_{Tu}$ and $H_{Tu}$ are the corresponding regularization parameter and the hidden layer output of the unlabeled samples in the target domain. Obviously, the update of the model is more complicated than ODAELM-S:

(16) $$\min_{\beta_{T}}\frac{1}{2}∥\beta_{T}{∥}^{2}+\frac{C_{T}}{2}∥t_{T}-H_{T}\beta_{T}{∥}^{2}+\frac{C_{Tu}}{2}{∥H_{Tu}\beta_{S}-H_{Tu}\beta_{T}∥}^{2}.$$

**Figure 3:**
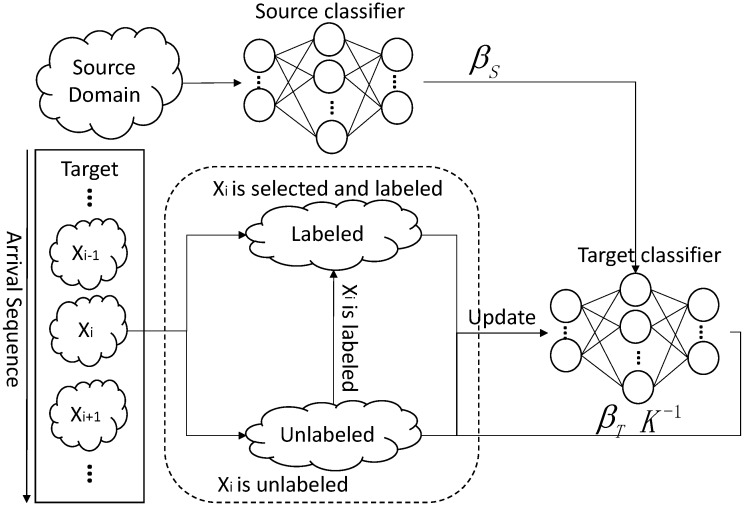
Online updating of ODAELM-T regarding different cases.

Figure 3 shows the procedure of ODAELM-T. Initially, a source classifier is trained on a source domain. Unlike ODAELM-S in which the classifier is built upon both source domain and labeled samples in the target domain, in DAELM-T, only the output weight matrix $\beta_{S}$ contributes to the initialization and updates of the target classifier. In ODAELM-T, solving Equation (16) results in two different cases depending on the numbers of rows and columns. In [27], when the number of rows in $H_{T}$ is smaller than that of columns, the Lagrange multiplier method was applied by using $\beta_{T}=H_{T}^{T}\alpha_{T}+H_{Tu}^{T}\alpha_{Tu}$. It is equal to assuming that the output weight is a linear combination of $H_{T}$ and $H_{Tu}$. However, due to the fact that the two cases are based on the rows and columns of $H_{T}$, it is reasonable to just assume $\beta_{T}=H_{T}^{T}\alpha_{T}$. Therefore, we can rewrite the output weight matrix of DAELM-T as Equation (17), where $P=H_{T}H_{T}^{T}$ and $Q=H_{T}H_{Tu}^{T}$.

(17) $$\beta_{T}=\left\{\begin{array}{c} {\left(I+C_{T}H_{T}^{T}H_{T}+C_{Tu}H_{Tu}^{T}H_{Tu}\right)}^{-1}\left(C_{T}H_{T}^{T}T_{T}+C_{Tu}H_{Tu}^{T}H_{Tu}\beta_{S}\right),\;H_{T}\;has\;more\;rows,\\ H_{T}^{T}{\left(I+C_{T}P+C_{Tu}P^{-1}QQ^{T}\right)}^{-1}\left(C_{T}T_{T}+C_{Tu}P^{-1}QH_{Tu}\beta_{S}\right),\;H_{T}\;has\;more\;columns. \end{array}\right.$$

Based on the appendix of [50], we know that the Gaussian kernel is of full rank in any case. In ELM, $HH^{T}$ and $H^{T}H$ are “ELM kernel” matrices [51]. Noting that Gaussian kernel is a special Radial Basis Function (RBF) kernel, we can ensure that $H_{T}H_{T}^{T}$ and $H_{T}^{T}H_{T}$ is of full rank if we use Gaussian function as the activation function. Moreover, we can further induce that Lemma A1 stands (see Appendix A), so Equation (17) can be transformed into online learning versions.

When new sample $X_{i}$ arrives, the algorithm determines whether to select and label a group of samples in the target domain. When no such process happens, $X_{i}$ is added into the unlabeled set. However, when the process takes place, δSet, which is described earlier, may consist of one or more samples and $X_{i}$ may or may not be in it. Due to the fact that manually labeling is time consuming, when $X_{i}$ arrives at first, it will be put into an unlabeled set. When δSet is determined, ODAELM-S will first take out the effects of these samples by using decremental learning in unlabeled sets. Subsequently, when the labeling is finished, ODAELM-T will perform incremental learning in the target domain. Therefore, there are three different learning mechanisms in ODAELM-T, which ensure the classifier is up-to-date during its lifetime.

The pseudo code for ODAELM-T is described as Algorithm 2. Initially, ODAELM-T generates a source classifier as ODAELM-S does (lines 2–3) and sets the labeled and unlabeled sets as LSet and USet, respectively. When new sample *x* in the target domain arrives, ODAELM-T calculates the probability of selecting and labeling samples in the target domain as ODAELM-S does (lines 5–6). When the selection and labeling happens, ODAELM-T firstly adds *x* into USet and selects the group of samples from target domain for labeling (lines 8–9). Note that, initially, there is no target classifier. Therefore, ODAELM-T will initialize an ELM with *L* hidden nodes when the first group of samples are labeled (lines 11–12). Once the target classifier is initialized, the target classifier will update itself based on the changes between LSet and USet. When *x* is added into USet, ODAELM-T follows unlabeled incremental learning. After a group of samples, i.e., δSet, are chosen, ODAELM-T will perform unlabeled decremental learning (lines 15–16). Subsequently, when δSet was manually labeled, ODAELM-T will perform incremental learning (lines 17–18). In the circumstance that no labeling happens, there is only unlabeled incremental learning (lines 21–22).

**Algorithm 2** Pseudo Code for ODAELM-T.**Input:**  L:= the number of hidden layer neurons;  Act:= the activation function type;  SD:= the source domain data; 1: Initialize labeled and unlabeled set as LSet and USet, respectively. 2: Initialize the source classifier of *L* hidden nodes using ActType with SD; 3: Let $H_{S}$ and $\beta_{S}$ be defined as Equation (17); 4: **while** new sample *x* in the target domain arrives **do** 5: Calculate the probability *P* for labeling; 6: Generate random value between 0 and 1 as *p*; 7: **if**
p<P
**then** 8:  Add *x* into USet; 9:  Select a group of samples as δSet in the target domain for labeling;10:  **if**
LSet is empty **then**11:   LSet=δSet;12:   Initialize a target classifier of *L* hidden nodes using Equation (17);13:  **else**14:   perform unlabeled incremental learning where increment is *x*;15:   USet=USet−δSet;16:    perform unlabeled decremental learning where decrement is δSet;17:   when the labeling process completes, LSet=LSet+δSet;18:   perform labeled incremental learning where increment is δSet;19:  **end if**20: **else**21:  Add *x* into USet;22:  perform unlabeled incremental learning where the increment is *x*;23: **end if**24: **end while**

#### 3.3.1. Unlabeled Incremental Learning

**Figure 4:**
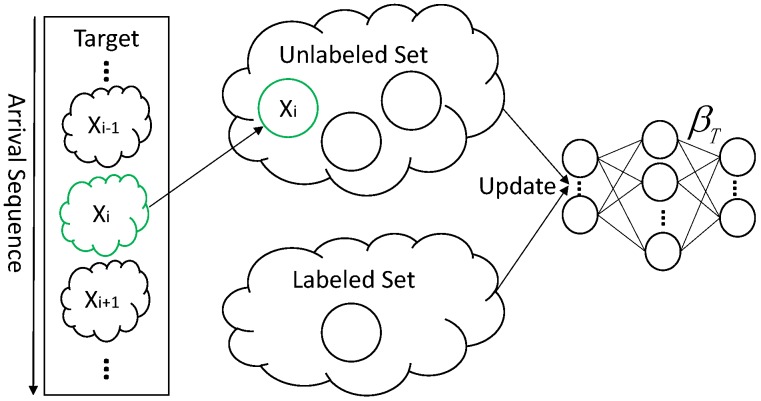
Unlabeled incremental learning.

As shown in Figure 4, only the unlabeled set changes by adding $X_{i}$ when a new sample arrives in an unlabeled incremental learning phase. The target classifier is calculated based on both of the samples in labeled and unlabeled sets. To provide efficient updates without repeatedly calculating the unchanged set, we can choose some intermediate result to compute the current output weight of the classifier.

For simple illustration purposes, we divided Equation (17) into two parts and let $Right=C_{T}H_{T}^{T}T_{T}+C_{Tu}H_{Tu}^{T}H_{Tu}\beta_{S}$. Let the intermediate result for current ELM be $K_{k}$, and the output weight matrix be $\beta_{T}^{k}=K_{k}^{-1}Right_{k}$. When new sample $X_{i}$ arrives, the corresponding hidden layer output of the target classifier can be computed as δh. Similar to OSELM, we can use the intermediate result $K_{k+1}^{-1}$ for (k+1)th update, where $K_{k+1}$ is defined as Equation (18):

(18) $$K_{k+1}=I+C_{T}H_{T}^{T}H_{T}+C_{Tu}\left(H_{Tu}^{T}H_{Tu}+\delta h^{T}\delta h\right)=K_{k}+C_{Tu}\delta h^{T}\delta h.$$

Subsequently, $Right_{k+1}$ becomes Equation (19):

(19) $$Right_{k+1}=Right_{k}+C_{Tu}\delta h^{T}\delta h\beta_{S}.$$

Based on the Sherman–Morrison–Woodbury formula, the inverse of $K_{k+1}$ can be obtained as Equation (20):

(20) $$K_{k+1}^{-1}={\left(K_{k}+C_{Tu}\delta h^{T}\delta h\right)}^{-1}=K_{k}^{-1}-C_{Tu}K_{k}^{-1}\delta h^{T}{\left(I+C_{Tu}\delta hK_{k}^{-1}\delta h^{T}\right)}^{-1}\delta hK_{k}^{-1}.$$

Note that $\beta_{T}^{k+1}=K_{k+1}^{-1}Right_{k+1}$. By multiplying $K_{k}K_{k}^{-1}$ before $Right_{k}$ in Equation (19), we can obtain the formula for $\beta_{T}^{k+1}$ as Equation (21):

(21) $$\begin{array}{cc} \beta_{T}^{k+1} & =K_{k+1}^{-1}\left(K_{k}K_{k}^{-1}Right_{k}+C_{Tu}\delta h^{T}\delta h\beta_{S}\right)\\ & =K_{k+1}^{-1}\left(K_{k}\beta_{T}^{k}+C_{Tu}\delta h^{T}\delta h\beta_{S}\right)\\ & =K_{k+1}^{-1}\left(\left(K_{k+1}-C_{Tu}\delta h^{T}\delta h\right)\beta_{T}^{k}+C_{Tu}\delta h^{T}\delta h\beta_{S}\right)\\ & =\beta_{T}^{k}-K_{k+1}^{-1}C_{Tu}\delta h^{T}\delta h\left(\beta_{T}^{k}-\beta_{S}\right). \end{array}$$

For the case where $H_{T}$ has more columns than rows, $K_{k+1}$ can be written as Equation (22). Let $Q_{k+1}Q_{k+1}^{T}$ and $Right_{k+1}$ be Equations (23) and (24), respectively:

(22) $$K_{k+1}=\left(I+C_{T}P+C_{Tu}P^{-1}Q_{k}Q_{k}^{T}+C_{Tu}P^{-1}H_{T}\delta h^{T}\delta hH_{T}^{T}\right)=K_{k}+C_{Tu}P^{-1}H_{T}\delta h^{T}\delta hH_{T}^{T},$$

(23) $$Q_{k+1}Q_{k+1}^{T}=H_{T}\left[\begin{array}{cc} H_{Tu}^{T} & \delta h^{T}, \end{array}\right]\left[\begin{array}{c} H_{Tu}\\ \delta h \end{array}\right]H_{T}^{T}=Q_{k}Q_{k}^{T}+H_{T}\delta h^{T}\delta hH_{T}^{T}$$

(24) $$Right_{k+1}=C_{T}T_{T}+C_{Tu}P^{-1}H_{T}\left(H_{Tu}^{T}H_{Tu}+\delta h^{T}\delta h\right)\beta_{S}=Right_{k}+C_{Tu}P^{-1}H_{T}\delta h^{T}\delta h\beta_{S}.$$

For illustration purposes, let $\delta \;k=\delta hH_{T}^{T}$. Similarly, $K_{k+1}^{-1}$ can be derived as Equation (25) based on the Sherman-Morrison-Woodbury formula:

(25) $$K_{k+1}^{-1}=K_{k}^{-1}-K_{k}^{-1}C_{Tu}P^{-1}{\left(I+\delta k^{T}\delta kK_{k}^{-1}C_{Tu}P^{-1}\right)}^{-1}\delta k^{T}\delta kK_{k}^{-1}.$$

Consequently, the output weight $\beta_{T}^{k+1}$ can be derived as Equation (26):

(26) $$\begin{array}{cc} \beta_{T}^{k+1} & =H_{T}^{T}K_{k+1}^{-1}Right_{k+1}\\ & =H_{T}^{T}K_{k+1}^{-1}\left(Right_{k}+C_{Tu}P^{-1}H_{T}\delta h^{T}\delta h\beta_{S}\right)\\ & =H_{T}^{T}K_{k+1}^{-1}\left(K_{k}K_{k}^{-1}Right_{k}+C_{Tu}P^{-1}H_{T}\delta h^{T}\delta h\beta_{S}\right)\\ & =\beta_{T}^{k}-C_{Tu}H_{T}^{T}K_{k+1}^{-1}P^{-1}H_{T}\delta h^{T}\delta h\left(\beta_{T}^{k}-\beta_{S}\right). \end{array}$$

#### 3.3.2. Unlabeled Decremental Learning

**Figure 5:**
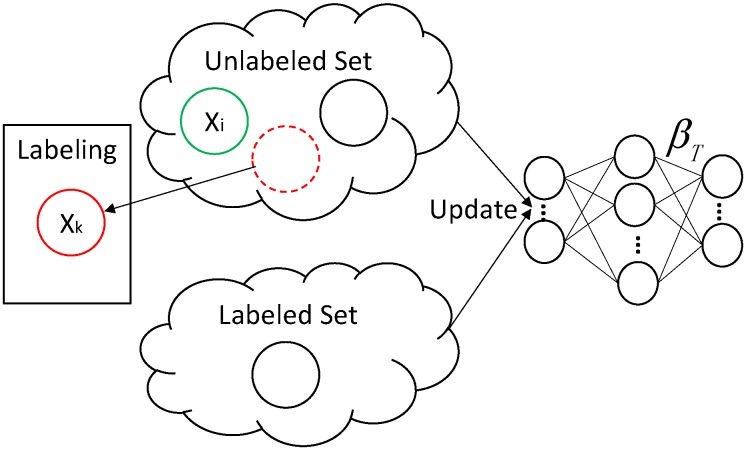
Unlabeled decremental learning.

When a group of samples (δSet) are selected for labeling, ODAELM-T updates the model first by eliminating the effects of samples in δSet. The process is called unlabeled decremental learning. As shown in Figure 5, $X_{k}$ is selected from an unlabeled set for labeling process. Note that *k* can be any arbitrary index from 1 to *i*, and there can be more than one sample for labeling.

Let the corresponding hidden layer output of δSet be δh. For the case where there are more rows than columns, let *K* and Right be written as Equation (27):

(27) $$\begin{array}{cc} K & =I+C_{T}H_{T}^{T}+C_{Tu}H_{Tu}H_{Tu},\\ Right & =C_{T}H_{T}^{T}H_{T}+C_{Tu}H_{Tu}^{T}H_{Tu}\beta_{S}. \end{array}$$

For current update procedure, the intermediate result $K_{k+1}^{-1}$ can be formulated as Equation (28):

(28) $$\begin{array}{cc} K_{k+1} & =K_{k}-C_{Tu}\delta h^{T}\delta h,\\ K_{k+1}^{-1} & =K_{k}^{-1}+C_{Tu}K_{k}^{-1}\delta h^{T}{\left(I-C_{Tu}\delta hK_{k}^{-1}\delta h^{T}\right)}^{-1}\delta hK_{k}^{-1}. \end{array}$$

Correspondingly, the output weight $\beta_{T}^{k+1}$ can be formulated as Equation (29):

(29) $$\beta_{T}^{k+1}=K_{k+1}^{-1}Right_{k+1}=\beta_{k}+C_{Tu}\delta h^{T}\delta h\left(\beta_{T}^{k}-\beta_{S}\right).$$

For the case where there are more columns than rows, let $P=H_{T}H_{T}^{T}$ and $Q=H_{T}H_{Tu}^{T}$. Since $H_{Tu}$ has changed, the corresponding results regarding *P* and *Q* can be written as Equation (30):

(30) $$\begin{array}{cc} {\left(H_{Tu}^{k+1}\right)}^{T}H_{Tu}^{k+1} & =H_{Tu}^{T}H_{Tu}-\delta h^{T}\delta h,\\ Q_{k+1}Q_{k+1}^{T} & =Q_{k}Q_{k}^{T}-H_{T}\delta h^{T}\delta hH_{T}^{T},\\ Q_{k+t}H_{Tu}^{k+1} & =H_{T}\left(H_{Tu}^{T}H_{Tu}-\delta h^{T}\delta h\right). \end{array}$$

Note that $K_{k+1}$ and $Right_{k+1}$ can be written as Equation (31):

(31) $$\begin{array}{cc} K_{k+1} & =K_{k}-C_{Tu}P^{-1}H_{T}\delta h^{T}\delta hH_{T}^{T},\\ Right_{k+1} & =Right_{k}-C_{Tu}P^{-1}H_{T}\delta h^{T}\delta h\beta_{S}. \end{array}$$

Subsequently, we can write the intermediate result and the output weight matrix as Equations (32) and (33), respectively, where δk is defined as $\delta k=\delta hH_{T}^{T}$:

(32) $$K_{k+1}^{-1}=K_{k}^{-1}+C_{Tu}K_{k}^{-1}P^{-1}{\left(I-C_{Tu}\delta k^{T}\delta kK_{k}^{-1}P^{-1}\right)}^{-1}\delta k^{T}\delta kK_{k}^{-1},$$

(33) $$\beta_{T}^{k+1}=H_{T}^{T}K_{k+1}^{-1}Right_{k+1}=\beta_{T}^{k}+C_{Tu}H_{T}^{T}K_{k+1}^{-1}P^{-1}H_{T}\delta h^{T}\delta h\left(\beta_{T}^{k}-\beta_{S}\right).$$

#### 3.3.3. Labeled Incremental Learning

**Figure 6:**
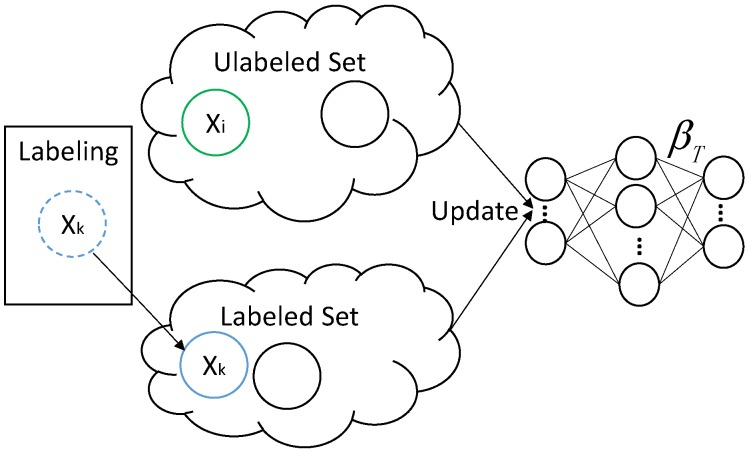
Labeled incremental learning.

After new samples are manually labeled, the incremental learning ensures that the model does not need to be recomputed from scratch. As shown in Figure 6, the unlabeled samples remain unchanged in this case. Therefore, the changes happens in $H_{T}$. The decremental part $X_{k}$ in this process is added into a labeled set. Note that $X_{k}$ in the figure is just an example and there can be more than one sample added into the labeled set.

Let the increment part be δSet with its label be δt, and the corresponding hidden layer output be δh. For the case where $H_{T}$ has more rows, let the current intermediate result $K_{k}$ be Equation (34):

(34) $$K_{k}=I+C_{T}H_{T}^{T}H_{T}+C_{Tu}H_{Tu}^{T}H_{Tu}.$$

When increment δSet arrives, the hidden layer output becomes $H_{T}^{k+1}=\left[\begin{array}{c} H_{T}\\ \delta h \end{array}\right]$ and the intermediate results can be derived as Equation (35):

(35) $$K_{k+1}=\left(I+C_{T}{\left(H_{T}^{k+1}\right)}^{T}H_{T}^{k+1}+C_{Tu}H_{Tu}^{T}H_{Tu}\right)=K_{k}+C_{T}\delta h^{T}\delta h.$$

By using Woodbury formula, the inverse of $K_{k+1}$ can be formulated as Equation (36):

(36) $$K_{k+1}^{-1}=K_{k}^{-1}-C_{T}K_{k}^{-1}\delta h^{T}{\left(I+C_{T}\delta hK_{k}^{-1}\delta h^{T}\right)}^{-1}\delta hK_{k}^{-1}.$$

Let Right be $C_{T}H_{T}^{T}T_{T}+C_{Tu}H_{Tu}^{T}H_{Tu}\beta_{S}$, and then $Right_{k+1}$ can be formulated as Equation (37):

(37) $$Right_{k+1}=Right_{k}+C_{T}\delta h^{T}\delta t.$$

Substitute Equations (36) and (37) into Equation (17), and the current output weight can be formulated as Equation (38):

(38) $$\beta_{T}^{k+1}=K_{k+1}^{-1}Right_{k+1}=K_{k+1}^{-1}\left(K_{k}\beta_{T}^{k+1}+C_{T}\delta h^{T}\delta t\right)=\beta_{T}^{k}-C_{T}K_{k+1}^{-1}\delta h^{T}\left(\delta h\beta_{T}^{k}-\delta t\right).$$

For the case where $H_{T}$ has more columns, let $K_{k}$ be Equation (39), where $P_{k}=H_{T}H_{T}^{T}$ and $Q_{k}=H_{T}H_{Tu}^{T}$:

(39) $$K_{k}=I+C_{T}P_{k}+C_{Tu}P_{k}^{-1}Q_{k}Q_{k}^{T}.$$

The inverse of $K_{k+1}$ involves updates of $P_{k+1}$ and $P_{k+1}^{-1}Q_{k+1}Q_{k+1}^{T}$. Meanwhile, the inverse of current $P_{k+1}$ becomes Equation (40) where $C=H_{T}\delta h^{T}$, $d=\delta h\delta h^{T}$ and $F_{2}={\left(d-CP_{k}^{-1}C\right)}^{-1}$:

(40) $$\begin{array}{cc} P_{k+1}^{-1} & ={\left[\begin{array}{cc} P_{k} & H_{T}\delta h^{T}\\ \delta hH_{T}^{T} & \delta h\delta h^{T} \end{array}\right]}^{-1}=\left[\begin{array}{cc} P_{k}^{-1}\left(I+CF_{2}C^{T}P_{k}^{-1}\right) & -P_{k}^{-1}CF_{2}\\ -F_{2}C^{T}P_{k}^{-1} & F_{2} \end{array}\right]=\left[\begin{array}{cc} P_{11} & P_{12}\\ P_{21} & P_{22} \end{array}\right]. \end{array}$$

Note that $Q_{k+1}Q_{k+1}^{T}$ and $P_{k+1}^{-1}Q_{k+1}Q_{k+1}^{T}$ can be formulated as Equations (41) and (42), respectively:

(41) $$\begin{array}{cc} Q_{k+1}Q_{k+1}^{T} & =\left[\begin{array}{c} H_{T}\\ \delta h \end{array}\right]H_{Tu}H_{Tu}^{T}{\left[\begin{array}{c} H_{T}\\ \delta h \end{array}\right]}^{T}=\left[\begin{array}{cc} H_{T}H_{Tu}^{T}H_{Tu}H_{T}^{T} & H_{T}H_{Tu}^{T}H_{Tu}\delta h^{T}\\ \delta hH_{Tu}^{T}H_{Tu}H_{T}^{T} & \delta hH_{Tu}^{T}H_{Tu}\delta h^{T} \end{array}\right]=\left[\begin{array}{cc} QQ_{11} & QQ_{12}\\ QQ_{21} & QQ_{22} \end{array}\right], \end{array}$$

(42) $$\begin{array}{cc} P_{k+1}^{-1}Q_{k+1}Q_{k+1}^{T} & =\left[\begin{array}{cc} PQQ_{11} & PQQ_{12}\\ PQQ_{21} & PQQ_{22} \end{array}\right]. \end{array}$$

To further compute the result, the formula becomes too complex, which increases the computation cost. Therefore, in this case, we simply update the output weight matrix $beta_{k+1}$ based on the batch learning version. However, in order for the two cases to combine together, we still use Equation (43) where $Right_{k+1}$ is formulated as Equation (44):

(43) $$\beta_{k+1}=\left[\begin{array}{c} H_{T}\\ \delta h \end{array}\right]K_{k+1}^{-1}Right_{k+1},$$

(44) $$\begin{array}{c} Right_{k+1}=C_{T}\left[\begin{array}{c} T_{T}\\ \delta t \end{array}\right]+C_{Tu}P_{k+1}^{-1}Q_{k+1}H_{Tu}\beta_{S}. \end{array}$$

Considering that $H_{T}$ changes with the arrival of $X_{i}$, the relation between the numbers of rows and columns in $H_{T}$ may not be static over time. To be specific, transitions may happen when the numbers of rows and columns are the same. Initially, the labeled set has few samples and increases as the labeling happens. Given enough time and samples, eventually, the size of the labeled set will match the size of the hidden neurons, i.e., the numbers of rows and columns in $H_{T}$ are the same. At this time stamp, case 1 and 2 coincide with each other. In order to continue performing incremental learning, the transition between the intermediate results of the two cases happens.

For the case where there are more rows than columns, $\beta_{T}=K_{1}^{-1}Right_{1}$ where $K_{1}$ and $Right_{1}$ can be formulated as Equation (45), respectively:

(45) $$\begin{array}{cc} K_{1} & =I+C_{T}H_{T}^{T}H_{T}+C_{Tu}H_{Tu}^{T}H_{Tu},\\ Right_{1} & =C_{T}H_{T}^{T}T_{T}^{T}+C_{Tu}H_{Tu}^{T}H_{Tu}\beta_{S}. \end{array}$$

For the case where there are more columns than rows, $\beta_{T}=K_{2}^{-1}Right_{2}$ where $K_{2}$ and $Right_{2}$ can be formulated as Equation (46):

(46) $$\begin{array}{cc} K_{2} & =I+C_{T}P+C_{Tu}P^{-1}QQ^{T},\\ Right_{2} & =C_{T}T_{T}+C_{Tu}P^{-1}QH_{Tu}\beta_{S}. \end{array}$$

When the rows are equal to the columns, the two expressions should be equal. In this case, both $H_{T}H_{T}^{T}$ and $H_{T}^{T}H_{T}$ are invertible. Hence, $H_{T}^{T}P^{-1}H_{T}={\left(H_{T}^{T}H_{T}\right)}^{-1}H_{T}^{T}H_{T}H_{T}^{T}P^{-1}H_{T}=I$. Therefore, intermediate results for transition between the two cases follow Equations (47) and (48):

(47) $$H_{T}^{T}Right_{2}=C_{T}H_{T}^{T}T_{T}+C_{Tu}H_{T}^{T}{\left(H_{T}H_{T}^{T}\right)}^{-1}H_{T}H_{Tu}^{T}H_{Tu}\beta_{S}=Right_{1},$$

(48) $$H_{T}^{T}K_{2}^{-1}H_{T}P^{-1}=K_{1}^{-1}.$$

## 4. Experiments

### 4.1. Experimental Data Description

In order to verify the effectiveness of the proposed methods, the chemical gas sensor dataset published on University of California Irvine (UCI) machine learning repository was used in the paper. Table 1 shows the details of the dataset. The data comprise readings from a sensor array of 16 metal gas sensors for continuous 36 months and one label field. For each gas sensor, the reading contains two steady status and six dynamic measurements. For details of the dataset, see [52].

### 4.2. Experimental Setup

All experiments in this paper were conducted in Matlab (2015b, MathWorks, Natick, MA, USA) on a Linux Workstation (Shanghai, China) with an E5 2.6-GHz CPU and 32 GB RAM. The settings for ELM network followed the work in [27], which used up to 1000 hidden layer nodes with RBF activation function. The parameters, tagged $C_{s}$, $C_{T}$ and $C_{Tu}$, have the same meaning and value as in [27].

The batches are organized in sequence based on their batch number. The current batch was used as a target domain, in which the samples were randomized and organized in sequence to simulate the arrival of samples in online scenarios. Meanwhile, the previous batch was used as the source domain. The performance of the target classifier was recoded at each arrival of samples and formulated as Equation (49), in which $Num_{+}$ was the number of correctly classified sample and Total referred to the number of the samples received so far:

(49) $$Accuracy=\frac{Num_{+}}{Total}.$$

To show the effectiveness of the proposed methods, we compared them with original batch learning versions, i.e., DAELM-T and DAELM-S. In addition, we chose an additional four commonly used machine learning-based methods. Firstly, ELM, SVM and Random Forest (RF) are chosen for their frequent uses in constructing classifiers, among which ELM shares a similar structure with the proposed methods while SVM and RF are two commonly used methods in classification. Secondly, ensemble based methods have been widely used in gas sensor domains. Hence, we also included Ensemble-SVM and Ensemble-ELM in which the sub-classifiers were built based on SVM and ELM, respectively. To sum up, we compared both ODAELM-S and ODAELM-T with seven machine learning-based methods. In the experiments, we did not use models such as Recurrent Neural Network (RNN) to learn the temporal behavior of the sensor errors. One reason is that the dataset we have at hand has been preprocessed by the publishers. The temporal information has been transformed into a steady and dynamic state of Exponential Moving Average (EMA) model, which does not include time information any more. Additionally, RNN and similar models such as Long Short-Term Memory (LSTM) are trained using Backpropagation through time (BPTT) or its variants. If the models are unfolded over time, they each can be viewed as a deep neural network. Training such deep neural networks can be very time-consuming, and so is updated. Hence, we do not compare the proposed methods with these models for the training time does not fit for online scenario. Accelerating the learning process of RNN or LSTM is another research area that is outside the scope of this paper.

In the experiments, we used the following experimental settings to evaluate the performances of the proposed methods regarding both classification accuracy and processing time.

- Setting 1: The labeled sets were selected from each target domain beforehand using the KS algorithm. In the arrival of the target samples, the methods treated the sample as labeled if it was in the pre-selected labeled sets, and vice versa. In this way, all the methods used in the experiment shared the same sample labeling process in the same arrival sequence of samples in the target domain. Although this setting does not work in practice, it can quantitatively evaluate the performance between offline and online learning, especially in processing time.
- Setting 2: The samples in the target domain was treated as a data flow and fed to the classification model in a one-by-one manner. The labeling process was treated as a random event in which the probability of labeling was related to current received and labeled samples only. To stop the the number of labeled samples from becoming too many, we set a threshold as 0.9. When the classification rate reaches the threshold, the probability of labeling is set to 0. This setting stimulates real application scenarios to provide qualitative comparisons for the proposed methods.
- Setting 3: The samples in the target domain was treated in the same way as setting 2. However, in this setting, we assume the testing accuracies on target domain would act as feedback and the probability of labeling was related to it as well. This setting stimulates another application scenario to show the effectiveness of the proposed methods.

### 4.3. Performance Evaluation

#### 4.3.1. Results Using Setting 1

To model the sequence of the sample, we randomized the data and used KS on them to select 50 representative samples for every target domain. Both recognition accuracies and the processing time were recorded each time when a new sample arrived.

**Figure 7:**
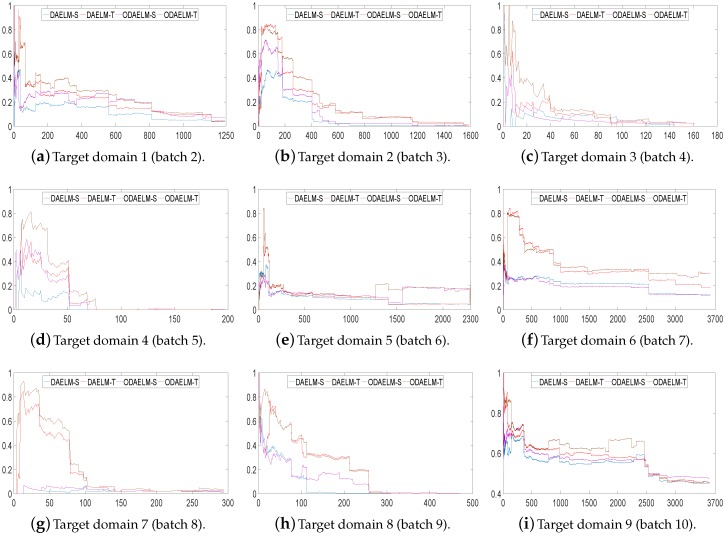
Classification errors on nine target domains using setting 1. The *y*-axis represents the error rate, in which 1 equals 100%. The *x*-axis is the number of samples arrived. The proposed methods and their batch learning versions are plotted in different colors.

Note that DAELM-S and DAELM-T do not work in online learning scenarios directly. In order to show the improvements of online learning versions, we used DAELM-S and DAELM-T to update the model and recorded the classification errors and processing time whenever a new sample arrived. As shown in Figure 7, the errors of four methods decrease as the number of samples increases. Meanwhile, the offline learning versions (DAELM-S and DAELM-T) have slightly better performances than their online learning versions (ODAELM-S and ODAELM-T), respectively. It is due to the computation errors accumulated whenever the updating happens. However, the difference between DAELM-S and ODAELM-S is minor and the same observation can be obtained between DAELM-T and ODAELM-T. In the meantime, the difference between DAELM-S and DAELM-T is notable. When the number of samples are limited, the error of DAELM-T increases by more than 10% when compared with DAELM-S, and similar results can be seen between ODAELM-T and ODAELM-S. When the number of samples increases, the performances of DAELM-S and DAELM-T draw close to each other and the difference is not large for observation except in the target domain 6 where ODAELM-T has around 10% less accuracy than ODAELM-S is. The same results apply to ODAELM-S and ODAELM-T. To sum up, the online learning versions have approximately equivalent performance compared with their offline learning versions regarding classification accuracy. In addition, ODAELM-S can work with limited samples while ODAELM-T reaches its maximum performance when the number is large.

**Figure 8:**
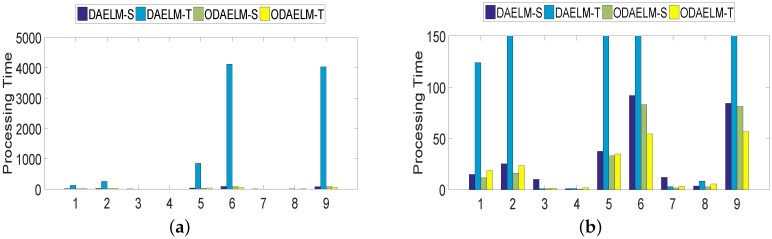
Processing time of DAELM-S, DAELM-T, ODAELM-S and ODAELM-T. The four methods are colored as the legend shows. (**a**) demonstrates the overall processing time for all nine target domains. Because DAELM-T is more time-consuming than the others; (**b**) is presented to help observe the differences of the other three methods.

To quantitatively show their computing complexities, the processing time of DAELM-S, DAELM-T, ODAELM-S and ODAELM-T were recorded and shown in Figure 8. In Figure 8a, the processing time of DAELM-T is remarkably larger than the other three methods. The reason is due to the fact that it updates whenever a new sample arrives regardless of labeling or not. Note that it follows a batch learning mechanism, which means that the computing complexity increases as the size of data increases. DAELM-S is also a batch learning method. The difference is that the updates of DAELM-S happen when a sample in the target domain is labeled. Therefore, much fewer updates happen compared with DAELM-T in general. In the experiment, there are 50 updates in total for DAELM-S. In fact, when the samples in the target domain are limited, such as target domains 7 and 8, the performance between DAELM-S and DAELM-T is almost indifferent. However, unlike DAELM-T whose computing complexity is closely tied to the size of target domain, the processing time of DAELM-S owes largely to the source domain, which is used to train the classifier. For example, in target domain 7, when the source domain has over 3000 samples, the processing time is almost two to three times that of target domain 8, which only uses less than 1000 samples as the source domain for training. As for ODAELM-S and ODAELM-T, it can be noted that both of the methods are, in general, less time-consuming than their batch learning versions. In some batches, the differences between ODAELM-S and DAELM-S are not large because the updates only happens 50 times. Nevertheless, for those target domain whose source domain is large, for example target domain 7, the difference between ODAELM-S and DAELM-S is obvious. As for ODAELM-T, it saves an enormous amount of time due to the online learning mechanism. Specifically, both ODAELM-T and DAELM-T have updates when a new sample arrives. However, ODAELM-T maintained the time for updates at a certain level regardless of the size of target domain while the updates of DAELM-T increase as the size of the received samples grows.

To better show the performance of the proposed methods, the average accuracies of the aforementioned methods and other commonly used classification algorithms are listed in Table 2 in which the letter D in the header represents the target domain. In the table, ELM, SVM and RF all include current labeled samples in the training process, i.e., the training set includes both source domain and the labeled sample in the target domain. En-ELM and En-SVM use the previous batches to train sub-classifiers and combine them together using their training accuracies as the weight. For example, in the target domain 3 (batch 4), batches 1 to 3 are used to train three sub-classifiers separately. As shown in the table, domain adaptation based methods are more accurate for all target domains in general. ELM, SVM and RF have over 90% and only DAELM-S and ODAELM-S exceed them by around 1%. However, ELM and SVM have relatively low accuracies in other target domains—for example in D8. RF has the second average accuracy, which is only less than 2% lower that of DAELM-S. However, the training part is even more time-consuming than DAELM-T. For example, in target domains 6 and 8, it took over 6000 s in total. For ensemble-based methods, i.e., En-ELM and En-SVM, their accuracies tend to be better than ELM and SVM when the target domain number is large. This is partly due to the fact that large target domain number means more source domains to generate sub-classifiers. In target domains 4, 5, 8 and 9, the ensemble-based methods outperform ELM and SVM. Between the online learning version, i.e., ODAELM-S and ODAELM-T, the performance of ODAELM-S is almost the same as DAELM-S, which has the highest average accuracy. ODAELM-T is around 8% lower that of DAELM-S and ODAELM-S in this setting. Considering the fact that, in setting 1, 50 representative samples are scattered in the arrival sequence, the reason for low accuracy may be the lack of enough labeled samples for DAELM-T and ODAELM-T.

As shown in Table 2, all methods experienced a large decrease of accuracy in D 9, given the fact that the data in all target domains except the 9th are collected in continuous months. For example, the data in the 1st target domain are collected from the 3rd to 10th months while the ones in the 2nd target domain are gathered from 11th to 13th. However, between target domains 8 and 9, there is a five-month vacuum. The limited number of samples in source domain may be another factor affecting the performance. However, given the classification accuracy in Figure 7, target domain 5, which has limited source domain samples, does not have such degradation in accuracy as target domain 9 does. Therefore, the number of samples in the target domain is not the reason, which leaves the long delay between target domains 8 and 9 being the main factor. Similar results can also be found in Table 3, Table 4, Table 5 and Table 6. The time of delay may have caused large distribution changes between target domains 8 and 9, which directly leads to the degradation of accuracies. However, we cannot be certain unless we can retrieve the data of the missing months.

#### 4.3.2. Results Using Setting 2

Note that, in practice, setting 1 does not exist since we cannot select the representative samples based on the distribution of the target domain beforehand. Therefore, to prove the effectiveness of the proposed methods, we use settings 2 and 3 to demonstrate possible labeling strategies in application scenarios.

**Figure 9:**
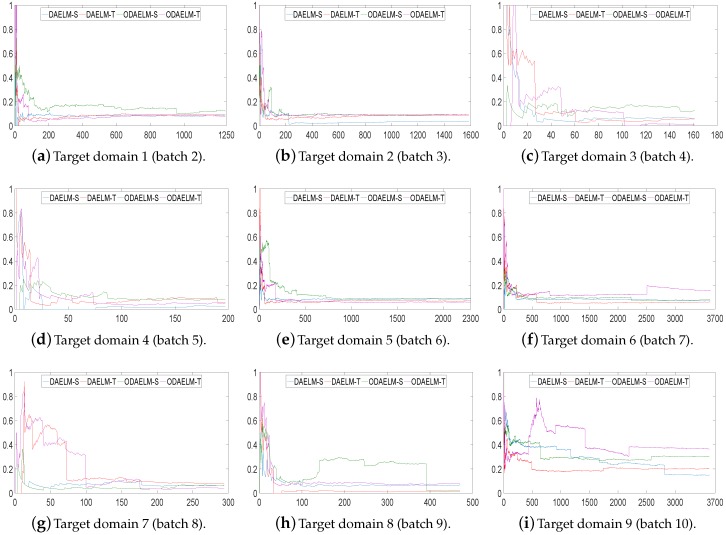
Classification errors on nine target domains using setting 2. The *x*- and *y*-axes are the same as Figure 7.

In setting 2, we choose the time of labeling and the number of labels based on the numbers of the current arrival and selected samples so far. Figure 9 shows the classification errors of nine target domains. As shown in the figure, all domain adaptation based methods quickly reach minimum errors for all target domains except for target domains 7 and 9. In target domain 7, both DAELM-T and ODAELM-T experience high classification errors before 1/3 of the samples arrives. This is partly due to the fact the number of the samples in this domain is less than that of other batches. Similar to the results in setting 1, ODAELM-S tends to work under limited labeled samples. In some circumstances, such as Figure 9h, patterns in the labeled set may contradict that in the source domain, which causes the error to increase between 150 to 400. Meanwhile, ODAELM-T does not have the problem and its errors keep decreasing as the number of labeled samples increases.

**Figure 10:**
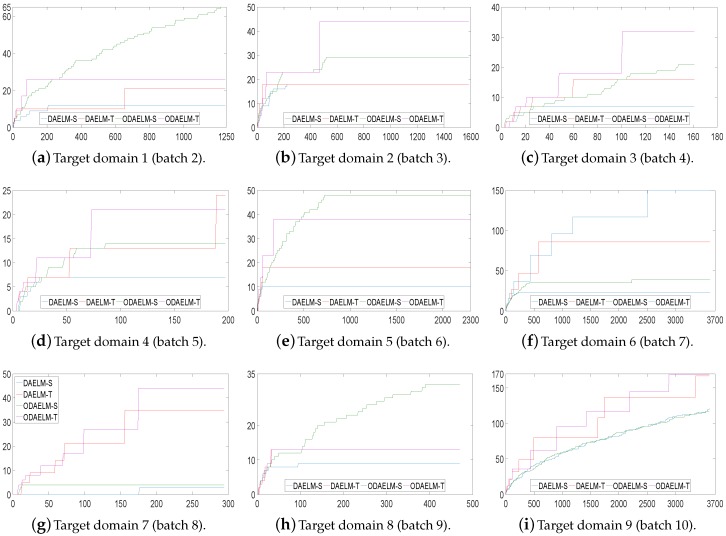
Numbers of labeled samples in nine target domains using setting 2. The *y*-axis is the number of labeled samples and the *x*-axis is the index of samples.

Figure 10 shows the corresponding numbers of labeled samples in nine target domains. As shown in the figure, the numbers of labeled samples for both offline and online learning methods are maintained in less than 65 except target domains 7 and 9 in which the total numbers of samples are over 3000. The reason is that the classification accuracy does not quickly reach the threshold. Hence, although the strategy ensures that the chance of labeling decreases as the number of labeled samples increases, the chance of labeling still exists and the value increases when new samples keep arriving and no labeling happens. However, even in these two domains, the labeled samples is less than 1/20 of the total samples.

Similar to setting 1, we further compared the performance of nine methods. Table 3 shows the average classification accuracies. Note that, unlike that in Table 2, RF in Table 3 is a batch learning that is trained like En-ELM and En-SVM. Even though the processing time can be omitted since RF is trained on a source domain, it cannot reach as high an accuracy as in setting 1. This is due to the drift phenomenon between domains. ODAELM-S and ODAELM-T in setting 2 have approximately the same performance on average. However, they are 3 to 4% less accurate than their batch learning versions. This is due to the accumulated computing errors over time. For specific batches, such as D2 and D3, the difference is around 0.2% to 0.8%. Note that, in setting 2, both DAELM-S and DAELM-T label more than 50 samples, which may cause even more time for updates. Considering the processing time saved by ODAELM-S and ODAELM-T, the drops in accuracies for the proposed online learning methods are still acceptable.

In Table 4, we further recorded the final classification error. Note that both ELM and SVM have large increments on average. This indicates that, although ELM and SVM reach their maximum classification accuracy, which is around 83%, the performance of the two methods over time is inferior to domain adaptation based methods. It can also be noted that the difference between offline and online learning versions of domain adaptation based methods are reduced to 2% to 3%. It indicates that the final classification accuracies between offline and online learning methods are close to each other when compared with the results in Table 3.

#### 4.3.3. Results Using Setting 3

In setting 3, the online sampling strategy uses the testing accuracies of the recognition model to help tune the selection of samples. To be more specific, the strategy tends to reduce the number of selected samples when the accuracy is maintained at a certain level.

**Figure 11:**
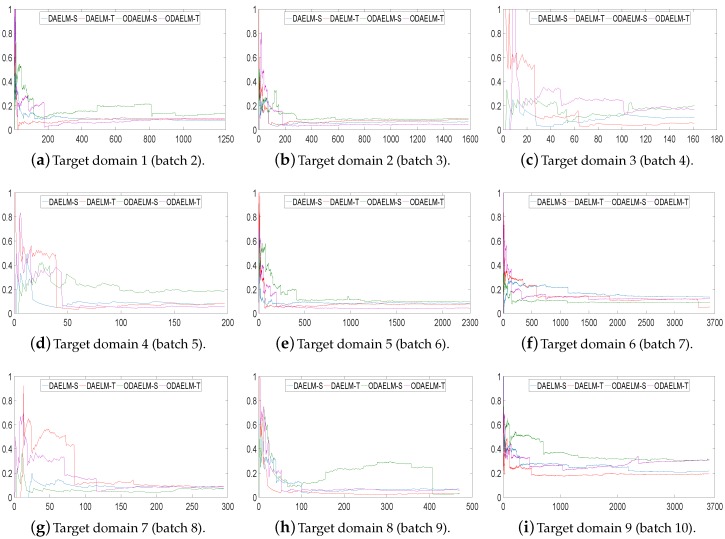
Classification errors on nine target domains using setting 3. The *x*- and *y*-axes are the same as Figure 7.

Figure 11 shows the performances of DAELM-S, DAELM-T, ODAELM-S and ODAELM-T in nine target domains. Similar to Figure 9, the accuracies of all four methods increase as the number of arrival sample increases. For most of the target domains, the four methods acted in a similar way as in setting 2, and reached their minimum error after around 1/3 of the samples arrived, which indicates that the two labeling strategies are both suitable for online learning. Compared with Figure 9, the accuracies of the proposed methods are maintained, which indicates that the online sampling strategy in setting 3 ensures the performance of ODAELM-S and ODAELM-T.

**Figure 12:**
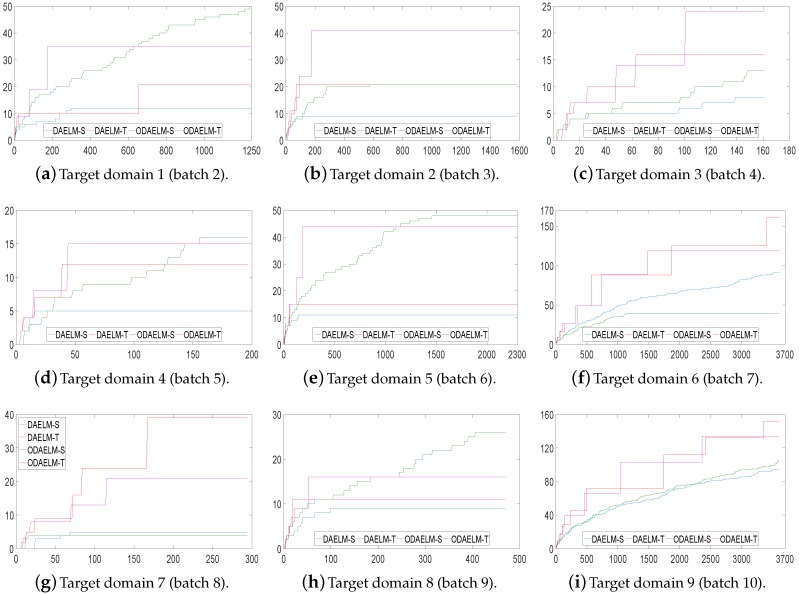
Numbers of labeled samples in nine target domains using setting 3. The *x*- and *y*-axes are the same as Figure 10.

To compare the number of selected samples in setting 3, we recorded the value in each update. Figure 12 shows the numbers of labeled samples. It can be noted that, compared with setting 2, all four methods use less samples in each target domain in general. The final numbers decrease by more than 10. In practice, the labeling costs not only time but also amount of human effort. With the testing error feedback, although the exact labels of the samples are still unknown, the four domain adaptation methods can save time by using less labeled sets. Between ODAELM-S and ODAELM-T, it can be seen that the former works with fewer selected samples while the other requires over 30 selected samples in general. If human resources for labeling the samples are limited, it is reasonable to choose ODAELM-S to work in practice. Nevertheless, there is still a chance that the increase of the labeled samples may decrease the accuracies of ODAELM-S, such as in Figure 11h. Therefore, ODAELM-T may be more suitable when there is a large number of samples labeled.

Table 5 shows the average classification accuracies in nine batches. Similar to setting 2, we compared the proposed methods with seven other algorithms. The ensemble-based methods and RF share the same procedure as in setting 2. Therefore, the results are the same. It can be noted that, in this setting, online learning versions still performed well and the classification accuracies were around 3% lower than their batch learning versions, which is the same as in setting 2. However, the differences are also acceptable considering the fact that online learning versions is less time-consuming.

The final accuracies shown in Table 6 also confirm the results in Table 5. Even though for some batches such as target domain 8 where DAELM-T labeled less samples than ODAELM-T, the difference value is not large, e.g., in the target domain 8 ODAELM-T labeled only eight more samples than DAELM-T. Even for target domain 5 where ODAELM-T labeled around 20 more samples, ODAELM-T is still more time-saving considering that the computing complexity of DAELM-T increases drastically as the size of the arrival sample increases.

## 5. Conclusions

In this paper, we proposed ODAELM-S and ODAELM-T for online sensor drift compensation in E-Nose systems. The proposed methods can update the model as new samples arrive, which is more time-saving compared with their batch learning versions. Meanwhile, we proposed two online labeling strategies to couple with the proposed methods.

Experiments on sensor drift dataset of six diverse compounds from 36 months demonstrate the effectiveness of the proposed methods regarding both classification accuracy and processing time. The results show that, under the same sampling and arrival sequences of the target domain, the proposed methods save more time than their batch learning versions do without losing the classification accuracy. In the meantime, the results under two online sampling strategies confirm the effectiveness of the proposed methods, which outperform the other classification algorithms. Between the two proposed methods, their capacities of identifying diverse gases draw close to each other eventually. However, ODAELM-S is more suitable to apply when the target domain is small and limit samples are labeled. ODAELM-T achieves its maximum capacity when the number of labeled samples is large, and outperforms ODAELM-S in specific target domains. In general, ODAELM-S is more feasible when the labeled samples are limited, while ODAELM-T can be used to replace ODAELM-S for better accuracies when the number of sample increases.

The online sampling strategies including the formula to calculate the probabilities of selecting and labeling samples in the target domain are the only two cases used in the paper. More sophisticated and accurate sampling models may be considered to improve the selection of representative samples. Meanwhile, human labor is a key factor in semi-supervised methods and the selection of representative samples may also be constrained by the factor, which is not included in the discussion of the paper. Future works may be extended to improve the sampling strategies under more restricted scenarios and parallel computing may be included to further reduce the processing time.

## Figures and Tables

**Table 1:** Dataset of sensor drift in E-Nose systems [52].

Batch No.	Months	Number of Samples					
		Ethanol	Ethylene	Ammonia	Acetaldehyde	Acetone	Toluene
1	1, 2	83	30	70	98	90	74
2	3, 4, 8, 9, 10	100	109	532	334	164	5
3	11, 12, 13	216	240	275	490	365	0
4	14, 15	12	30	12	43	64	0
5	16	20	46	63	40	28	0
6	17, 18, 19, 20	110	29	606	574	514	467
7	21	360	744	630	662	649	568
8	22, 23	40	33	143	30	30	18
9	24, 30	100	75	78	55	61	101
10	36	600	600	600	600	600	600

**Table 2:** Comparisons of average accuracies using setting 1.

Methods	Average Accuracy (%)									
	D 1	D 2	D 3	D 4	D 5	D 6	D 7	D 8	D 9	Average
ELM	63.6	52.5	69.9	64.1	64.2	70.7	91.9	49.7	25.2	61.3
SVM	80.8	84.2	88.9	86.8	66.6	76.6	95.3	69.9	34.2	75.9
RF	82.4	89.2	97.2	97.6	83.7	72.6	95.5	92.5	43.9	83.8
En-ELM	76.9	74.0	67.4	97.7	72.2	69.9	84.1	41.1	46.6	70.0
En-SVM	76.8	74.2	82.6	68.0	66.5	66.1	53.7	59.0	48.6	66.2
DAELM-S	88.2	81.7	94.1	96.5	90.9	79.5	97.9	92.5	45.9	85.2
DAELM-T	79.7	80.9	89.9	90.8	89.5	64.5	83.0	79.0	42.6	77.8
ODAELM-S	81.3	87.8	94.0	90.2	85.8	81.2	96.9	89.1	45.6	83.5
ODAELM-T	75.5	79.9	84.0	85.3	83.3	62.0	78.6	77.6	40.8	74.1

**Table 3:** Comparisons of average classification accuracies using setting 2.

Methods	Average Accuracy (%)									
	D 1	D 2	D 3	D 4	D 5	D 6	D 7	D 8	D 9	Average
ELM	84.5	64.3	47.7	87.4	91.5	78.5	88.8	89.7	71.4	78.2
SVM	78.6	87.7	64.9	57.8	85.8	81.7	55.3	87.0	61.4	73.4
RF	61.4	64.9	67.7	62.9	53.5	65.9	94.2	39.3	21.4	59.0
En-ELM	76.9	74.0	67.4	97.7	72.2	69.9	84.1	41.1	46.6	70.0
En-SVM	76.8	74.2	82.6	68.0	66.5	66.1	53.7	59.0	48.6	66.2
DAELM-S	91.7	95.7	88.9	95.1	91.5	91.4	93.2	92.2	71.9	90.2
DAELM-T	91.2	91.9	85.1	89.7	92.6	92.9	82.2	96.2	79.0	89.0
ODAELM-S	84.0	90.0	85.7	89.2	87.5	90.6	94.9	80.6	68.5	85.7
ODAELM-T	91.5	90.2	87.5	90.3	92.5	85.3	81.2	89.2	58.6	85.4

**Table 4:** Comparisons of final classification accuracies using setting 2.

Methods	Final Accuracy (%)									
	D 1	D 2	D 3	D 4	D 5	D 6	D 7	D 8	D 9	Average
ELM	90.4	71.4	57.8	93.4	93.6	84.2	88.3	90.4	77.6	83.0
SVM	87.8	90.5	65.8	86.3	95.0	90.1	58.8	97.0	73.3	82.7
RF	63.3	64.8	66.5	58.9	51.5	66.0	92.5	39.6	21.8	58.4
En-ELM	76.9	82.5	74.5	97.5	72.3	69.5	85.4	46.0	52.9	73.0
En-SVM	73.2	74.8	86.9	70.1	65.6	65.5	54.4	62.3	48.6	60.2
DAELM-S	91.3	96.5	93.8	97.5	91.5	92.7	93.9	93.2	85.0	92.8
DAELM-T	90.6	91.3	94.4	94.4	92.9	93.9	92.2	98.7	79.8	92.0
ODAELM-S	87.7	90.8	87.0	91.9	91.1	92.1	93.5	97.9	69.9	89.1
ODAELM-T	92.2	90.7	98.8	94.9	94.0	84.4	95.9	92.3	63.7	89.7

**Table 5:** Comparisons of average classification accuracies using setting 3.

Methods	Average Accuracy (%)									
	D 1	D 2	D 3	D 4	D 5	D 6	D 7	D 8	D 9	Average
ELM	75.3	60.4	45.5	91.6	90.7	73.6	85.6	84.3	66.8	74.9
SVM	78.6	88.6	64.5	61.4	85.7	81.5	55.3	85.1	61.2	73.5
RF	61.4	64.9	67.7	62.9	53.5	65.9	94.2	39.3	21.4	58.4
En-ELM	76.9	74.0	67.4	97.7	72.2	69.9	84.1	41.1	46.6	70.0
En-SVM	76.8	74.2	82.6	68.0	66.5	66.1	53.7	59.0	48.6	66.2
DAELM-S	90.3	93.4	90.6	90.2	91.2	82.8	91.4	90.9	74.5	88.4
DAELM-T	91.2	92.0	84.9	85.0	91.9	85.5	80.0	94.8	80.0	87.3
ODAELM-S	83.0	89.5	83.6	78.3	86.4	90.0	93.9	79.5	64.1	83.1
ODAELM-T	90.5	93.5	76.9	87.4	94.0	85.8	84.3	90.3	71.7	86.0

**Table 6:** Comparisons of final classification accuracies using setting 3.

Methods	Final Accuracy (%)									
	D 1	D 2	D 3	D 4	D 5	D 6	D 7	D 8	D 9	Average
ELM	81.1	63.6	51.6	96.4	95.6	78.5	90.5	94.0	70.4	80.2
SVM	87.8	93.6	65.8	85.8	94.8	90.1	58.8	94.0	73.6	82.1
RF	63.3	64.8	66.5	58.9	51.5	66.0	92.5	39.6	21.8	58.4
En-ELM	76.9	82.5	74.5	97.5	72.3	69.5	85.4	46.0	52.9	73.0
En-SVM	73.2	74.8	86.9	70.1	65.6	65.5	54.4	62.3	48.6	60.2
DAELM-S	92.2	93.5	90.1	91.9	91.7	86.5	92.5	93.2	78.3	89.9
DAELM-T	90.6	91.4	94.4	91.9	92.1	94.7	91.5	97.0	80.7	91.6
ODAELM-S	86.8	90.7	80.0	81.2	90.5	90.8	92.9	97.2	68.7	86.5
ODAELM-T	91.7	95.4	83.2	94.4	95.6	87.9	90.8	94.0	69.1	89.1

## Abbreviations

The following abbreviations are frequently used in this manuscript:

KS	Kennard-Stone algorithm
RF	Random Forest
ELM	Extreme Learning Machine
SVM	Support Vector Machine
En-ELM	Ensemble based on ELM
En-SVM	Ensemble based on SVM
DAELM	Domain Adaptation Extreme Learning Machine
DAELM-S	Source Domain Adaptation Extreme Learning Machine
DAELM-T	Target Domain Adaptation Extreme Learning Machine
ODAELM-S	Online Source Domain Adaptation Extreme Learning Machine
ODAELM-T	Online Target Domain Adaptation Extreme Learning Machine

## Appendix A

**Lemma** **A1.** If $C_{T},C_{Tu}>0$, $H_{T}\in R^{n_{1}*l}$ and $H_{Tu}\in R^{n_{2}*l}$ are any arbitrary matrices defined in the paper, then $I+C_{T}P+C_{Tu}P^{-1}H_{T}H_{Tu}^{T}H_{Tu}H_{T}^{T}$ has an inverse where $P=H_{T}H_{T}^{T}$.

**Proof.** Let *A* be defined as $A=I+C_{T}P+C_{Tu}P^{-1}H_{T}H_{Tu}^{T}H_{Tu}H_{T}^{T}$. Consider a sequence of rank-one updates of *A* as $A_{k}=I+C_{T}P+C_{Tu}P^{-1}H_{T}\left(\sum_{i=0}^{k}h_{i}^{T}h_{i}\right)H_{T}^{T}$, where $h_{i}\in R^{1*l}$ is the *i*th row of $H_{Tu}$. If we can prove $A_{k}$ has inverse for any arbitrary column vector $h_{i}$, Lemma A1 is proved. Let $A_{k}$ be written as Equation (A1). Let $c_{k}=C_{Tu}P^{-1}H_{T}h_{i}$ and $d_{k}^{T}=h_{i}H_{T}^{T}$. Based on generalized inverse theory [53], the generalized inverse of $A_{k}$ has a unique form as Equation (A2) if $A_{k-1}$ has inverse and Equation (A3) satisfies. It is easy to verify that, in this case, the generalized inverse of $A_{k-1}$ is the inverse:

(A1) $$A_{k}=A_{k-1}+C_{Tu}P^{-1}H_{T}h_{i}^{T}h_{i}H_{T}^{T},$$

(A2) $$A_{k}^{†}=A_{k-1}^{-1}-\frac{A_{k-1}^{-1}c_{k}d_{k}^{T}A_{k-1}^{-1}}{1+d_{k}^{T}A_{k-1}c_{k}},$$

(A3) $$1+d_{k}^{T}A_{k-1}^{-1}c_{k}\neq 0.$$

Therefore, the problem becomes proving that $A_{k-1}$ has inverse and Equation (A3) stands. To achieve the goal, we can prove a stronger case as Equation (A4):

(A4) $$d_{k}^{T}A_{k-1}^{-1}c_{k}\geq 0\;if\;A_{k-1}^{-1}\;exist.$$

Let $A_{0}=I+C_{T}H_{T}H_{T}^{T}$ and *h* be an arbitrary row of $H_{Tu}$. Note that $C_{T}>0$, so $A_{0}$ has an inverse. Based on Woodbury’s formula, we can write $A_{0}^{-1}$ as Equation (A5):

(A5) $$A_{0}^{-1}=I-C_{T}H_{T}{\left(I+C_{T}H_{T}^{T}H_{T}\right)}^{-1}H_{T}^{T}.$$

Let $A_{1}=A_{0}+c_{1}d_{1}^{T}$, where $c_{1}=C_{Tu}P^{-1}H_{T}h^{T}$ and $d_{1}^{T}=hH_{T}^{T}$. Subsequently, we can write $d_{1}^{T}A_{0}^{-1}c_{1}$ as Equation (A6) by using Woodbury’s formula. Note that $C_{T}H_{T}^{T}H_{T}$ is a positive semi-definite, so it is unitarily similar to a diagonal matrix, i.e., $H_{T}^{T}H_{T}=Udiag\left(\epsilon_{1},\epsilon_{2}\ldots ,\epsilon_{y}\right)U^{T},$ where $\epsilon_{1}>\epsilon_{2}>\ldots >\epsilon_{y}>0$. Similarly, $I+C_{T}H_{T}H_{T}^{T}$ is unitarily similar to a diagonal matrix, i.e., $I+C_{T}H_{T}H_{T}^{T}=Udiag\left(1+C_{T}\epsilon_{1},1+C_{T}\epsilon_{2}\ldots ,1+C_{T}\epsilon_{y}\right)U^{T}$. Note that they share the same *U*; therefore, $d_{1}^{T}A_{0}^{-1}c_{1}\geq 0$ stands. Consequently, $A_{1}$ has an inverse:

(A6) $$\begin{array}{cc} d_{1}^{T}A_{0}^{-1}c_{1} & =hH_{T}^{T}A_{0}^{-1}C_{Tu}P^{-1}H_{T}h^{T}\\ & =C_{Tu}hH_{T}^{T}\left(I-C_{T}H_{T}{\left(I+C_{T}H_{T}^{T}H_{T}\right)}^{-1}H_{T}^{T}\right)P^{-1}H_{T}h^{T}\\ & =C_{Tu}h\left(I-C_{T}H_{T}^{T}H_{T}{\left(I+C_{T}H_{T}^{T}H_{T}\right)}^{-1}\right)H_{T}^{T}P^{-1}H_{T}h^{T}\\ & =C_{Tu}h{\left(I-C_{T}H_{T}^{T}H_{T}\right)}^{-1}H_{T}^{T}P^{-1}H_{T}h^{T}\\ & =C_{Tu}h\left(I-C_{T}H_{T}^{T}{\left(I+C_{T}H_{T}H_{T}^{T}\right)}^{-1}H_{T}\right)H_{T}^{T}P^{-1}H_{T}h\\ & =C_{Tu}hH_{T}^{T}\left(P^{-1}-C_{T}{\left(I+C_{T}H_{T}H_{T}^{T}\right)}^{-1}\right)H_{T}h^{T}\\ & =C_{Tu}hH_{T}^{T}P^{-1}{\left(I+C_{T}H_{T}H_{T}^{T}\right)}^{-1}H_{T}h^{T}\\ & =C_{Tu}hH_{T}^{T}P^{-1}A_{0}^{-1}H_{T}h^{T}. \end{array}$$

Based on Equation (A6), we can further define Equation (A7), which is inversible:

(A7) $$B_{0}=A_{0}^{-1}P^{-1}.$$

Assume $A_{k}$ has an inverse. By using Woodbury’s formula, we can write $A_{k}^{-1}$ as Equation (A8):

(A8) $$\begin{array}{cc} A_{k}^{-1} & ={\left(A_{0}+C_{Tu}P^{-1}H_{T}H_{Tu}^{T}H_{Tu}H_{T}\right)}^{-1}\\ & =A_{0}^{-1}-C_{Tu}A_{0}^{-1}P^{-1}H_{T}H_{Tu}^{T}{\left(I+C_{Tu}H_{Tu}B_{0}H_{Tu}^{T}\right)}^{-1}H_{Tu}H_{T}^{T}A_{0}^{-1}. \end{array}$$

For the case of $A_{k+1}=A_{k}+C_{Tu}P^{-1}H_{T}h^{T}hH_{T}^{T}$ where *h* is an arbitrary row of $H_{Tu}$, we can combine Equations (A7) and (A8) to write $d_{k+1}^{T}A_{k}^{-1}c_{k+1}$ as Equation (A9), where $c_{k+1}=C_{Tu}P^{-1}H_{T}h^{T}\in R^{n_{1}*1}$ and $d_{k+1}^{T}=hH_{T}^{T}\in R^{1*n_{1}}$. Note that *h* is an arbitrary row of $H_{Tu}$. Similarly, we have $d_{k+1}^{T}A_{k}^{-1}c_{k+1}\geq 0$ stands:

(A9) $$\begin{array}{cc} d_{k+1}^{T}A_{k}^{-1}c_{k+1} & =C_{Tu}hH_{T}^{T}\left(B_{0}-C_{Tu}B_{0}H_{Tu}^{T}{\left(I+C_{Tu}H_{Tu}B_{0}H_{Tu}^{T}\right)}^{-1}H_{Tu}B_{0}\right)H_{T}h^{T}\\ & =C_{Tu}h{\left(B_{0}^{-1}+C_{Tu}H_{Tu}^{T}H_{Tu}\right)}^{-1}h^{T}. \end{array}$$

By using mathematical induction, we can prove that $d_{k}^{T}A_{k-1}^{-1}c_{k}\geq 0$ stands for any *k*. Subsequently, $A_{k}$ has inverse for any *k*. To sum up, Lemma A1 has been proved. ☐
